# Draft genomes of four *Kluyveromyces marxianus* isolates retrieved from the elaboration process of henequen (*Agave fourcroydes*) mezcal

**DOI:** 10.1128/mra.00861-23

**Published:** 2024-01-31

**Authors:** Luis Lozano-Aguirre, Morena Avitia, Patricia Lappe-Oliveras, Cuauhtémoc Licona-Cassani, Miguel A. Cevallos, Sylvie Le Borgne

**Affiliations:** 1 Unidad de Análisis Bioinformáticos, Centro de Ciencias Genómicas, Universidad Nacional Autónoma de México, Cuernavaca, Mexico; 2 Laboratorio Nacional de Ciencias de la Sostenibilidad (LANCIS), Instituto de Ecología, Universidad Nacional Autónoma de México, Ciudad de México, Mexico; 3 Laboratorio de Micromicetos, Instituto de Biología, Universidad Nacional Autónoma de México, Ciudad de México, Mexico; 4 Laboratorio de Genómica Industrial, Escuela de Ingeniería y Ciencias, Tecnológico de Monterrey, Monterrey, Mexico; 5 Programa de Genómica Evolutiva, Centro de Ciencias Genómicas, Universidad Nacional Autónoma de México, Cuernavaca, Mexico; 6 Departamento de Procesos y Tecnología, Universidad Autónoma Metropolitana-Unidad Cuajimalpa, Ciudad de México, Mexico; University of California Riverside, Riverside, California, USA

**Keywords:** *Kluyveromyces marxianus*, agave, biodiversity

## Abstract

We report the draft genomes of four *Kluyveromyces marxianus* isolates obtained from the elaboration process of henequen (*Agave fourcroydes*) mezcal, a Mexican alcoholic beverage. The average nucleotide identity analysis revealed that isolates derived from agave plants are distinct from those from other environments, including agave fermentations.

## ANNOUNCEMENT

*Kluyveromyces marxianus* is a thermotolerant yeast with a fast growth rate and the ability to metabolize a wide range of carbohydrates making it a promising cell factory for industrial biotechnology (1–3). *K. marxianus* has been frequently isolated from dairy products (4) and other habitats such as fermented beverages (5), plants and fruits (6, 7), and sugarcane mills (8, 9), among others. Isolates from agave and associated fermentations may constitute a new clade within the *K. marxianus* species (4). Among the 21 *K*. *marxianus* genomes available in NCBI only two correspond to strains from agave: UFS-Y2791 from an agave plant (Schabort, D. T., Letebele, P. K., Steyn, L., Kilian, S. G. and duPreez, J. C., unplished data) and SLP1 from spontaneous mezcal fermentation (5). Here, we present the draft genomes of four *K. marxianus* isolates isolated from the elaboration process of henequen mezcal as previously described (10). Henequen (*Agave fourcroydes*) is an agave species native to the Yucatan Peninsula.

DNA was prepared from overnight cultures in yeast extract-peptone-dextrose broth at 30°C and 150 rpm (10) using the Quick-DNA Fungal/Bacterial Miniprep Kit (Zymo Research) following the manufacturer’s instructions. DNA quality and purity were assessed by 0.7% (wt/vol) agarose gel electrophoresis in 1× TBE buffer, and UV absorbance measurements were performed on a Nanodrop 2000 spectrophotometer (Thermo Scientific). DNA was quantified using a Qubit 3.0 ﬂuorometer (Life Technologies). Paired-end genomic DNA libraries were constructed using the TruSeq Nano kit (Illumina) according to the manufacturer’s instructions. Libraries’ quality and quantity were verified using a 2100 BioAnalyzer (Agilent Technologies). Sequencing was performed on the Illumina HiSeq 2500 platform through the standard rapid-sequencing protocol to generate 150-bp paired-end reads.

Reads’ quality was assessed with FastQC v0.11.9 (11). The adapters and low-quality bases were discarded using Trimmomatic v.0.39 with default parameters (12). *De novo* genome assemblies were generated using Velvet v.1.2.10 (kmer 37) (13) and Spades v.3.12.0 (kmers 21, 33, 55, 77, and 99) (14), and the obtained assemblies were merged with Metassembler v.1.5 using the Spades contigs as primary assembly (15). Assemblies’ quality was assessed using QUAST v.4.1 (16). Gene prediction was performed with Funannotate (v.1.8.14) using *Kluyveromyces lactis* as the training species (17). Assemblies’ completeness was evaluated with BUSCO v.5.4.7 using the saccharomycetes_odb10 database (18). Average nucleotide identity (ANI) analysis was calculated with pyani v0.2 using the ANIb method (19). The heatmap was built in R with ggplot2 and pheatmap.

**Fig 1:**
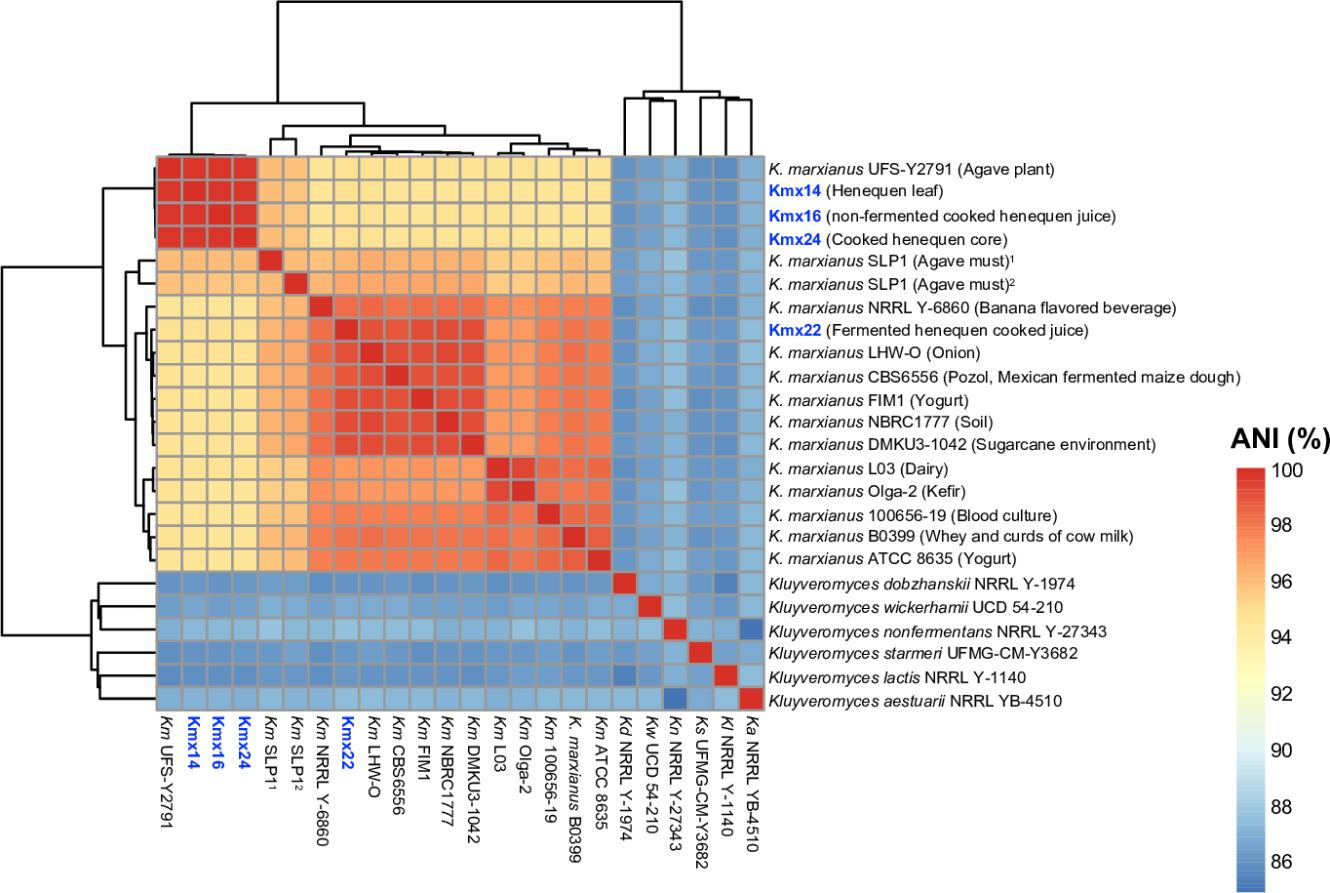
Heatmap of ANI values. The isolates sequenced here are indicated in blue color. The *Kluyveromyces* genomes were downloaded from NCBI: *K. marxianus* UFS-Y2791 (GenBank accession number: GCA_001692465.1), *K. marxianus* SLP1 alternate-pseudohaplotype (GCA_021014395.1), *K. marxianus* SLP1 principal pseudohaplotype of diploid (GCA_021014425.1), *K. marxianus* NRRL Y-6860 (GCA_002356615.1), *K. marxianus* LHW-O (GCA_003046155.1), *K. marxianus* CBS6556 (GCA_016625955.1), *K. marxianus* FIM1 (GCA_001854445.2), *K. marxianus* NBRC 1777 (GCA_001417835.1), *K. marxianus* DMKU3-1042 (GCA_001417885.1), *K. marxianus* L03 (GCA_008000265.1), *K. marxianus* Olga-2 (GCA_016584165.1), *K. marxianus* 100656–19 (GCA_902364165.1), *K. marxianus* B0399 (GCA_001660455.1), *K. marxianus* ATCC 8635 (GCA_017309885.1), *K. dobzhanskii* NRRL Y-1974 (GCA_003705805.2), *K. wickerhamii* UCD 54–210 (GCA_000179415.1), *K. nonfermentans* NRRL Y-27343 (GCA_003670155.1), *K. starmeri* UFMG-CM-Y3682 (GCA_008973615.1), *K. lactis* NRRL Y-1140 (GCA_000002515.1), and *K. aestuarii* NRRL YB-4510 (GCA_003707555.1).

Table 1 details the sequencing data, assemblies’ statistics, BUSCO scores, and ANI values. Isolates UFS-Y2791, Kmx14, Kmx16, and Kmx24 from agave plant, henequen leaf, non-fermented henequen cooked juice, and cooked henequen core formed a separate group with ANI values greater than 99% between each other (Fig. 1). Interestingly, isolates Kmx22 and SLP1 from fermented henequen cooked juice and mezcal fermentations, respectively, did not belong to this group and exhibited more relatedness to *K. marxianus* isolates from dairy and other environments. These data confirm that there is further yeast diversity to be accessed in agave environments (4) in a similar way to what has been described for cactus yeasts (20).

**TABLE 1:** General features of the sequenced genomes

Isolate	Origin	No. of reads	No. of contigs	Total length (Mb)	Coverage (×)	GCcontent (%)	*N*_50_ (kb)	*L*_50_ (kb)	BUSCO scores (%)		ANI score (%)^*c*^	ANI coverage (%)^*c*^	No. of predicted genes	No. of proteins	GenBank assembly accession no.	SRA accession no.
									C*^a^*	D^*b*^						
Kmx14	Henequen leaf	4,395,815	215	10.6	116	39.99	87,645	37	98.9	0.3	94.5	94.9	4,923	4,744	GCA_029873725.1	SRR23105050
Kmx16	Non-fermented henequen cooked juice	4,686,443	219	10.5	124	40.04	87,096	36	98.7	0.1	94.6	94.2	4,916	4,747	GCA_029873675.1	SRR23105048
Kmx22	Fermented henequen cooked juice	5,405,732	396	10.6	142	40.11	45,399	74	99.4	0.1	99.2	96.9	4,935	4,773	GCA_029873665.1	SRR23105047
Kmx24	Cooked henequen core	3,024,326	218	10.6	79	39.99	78,647	40	99.1	0.1	94.5	95	4,922	4,743	GCA_029873655.1	SRR23105046

^*a*^ C, completeness.

^*b*^ D, duplication level.

^*c*^ ANI against the reference genome *K. marxianus* DMKU3-1042.

## Data Availability

The genome assembly generated in this study and the reads are deposited under BioProject ID PRJNA904382 at the NCBI.
